# Isomers of *β*,*β*-Dinitro-5,10,15,20-tetraphenylporphyrin Derivatives: Valuable Starting Materials for Further Transformations

**DOI:** 10.3390/molecules24050838

**Published:** 2019-02-27

**Authors:** Agnieszka Mikus, Mariusz Rosa, Stanisław Ostrowski

**Affiliations:** Faculty of Chemistry, Warsaw University of Technology, ul. Noakowskiego 3, 00-664 Warszawa, Poland; mariuszrosa1@gmail.com (M.R.); stan@ch.pw.edu.pl (S.O.)

**Keywords:** porphyrins, complexes, *β*-nitration, electrophilic substitution, nitric acid, 2D NMR.

## Abstract

The synthesis, chromatographic isolation, and structure elucidation of *β*,*β*-substituted isomers of dinitro-5,10,15,20-tetraphenylporphyrin complexes are described. *meso*-Tetraphenyl-porphyrin chelates (Cu^II^, Ni^II^, Co^II^) upon reaction wit e.g., nitric acid (yellow HNO_3_, *d* = 1.52, diluted to 25–50%) in CHCl_3_ formed a mixture of nitro-derivatives with combined yields of *ca* 80%. This nitration (under optimized conditions: 25–30% HNO_3_, 30–40 min, r.t.) can be carried out selectively to give mainly *β*,*β*-dinitro-compounds in yields of up to 73%. From the above mixtures of five possible regioisomers that can be formed, usually two or three of them were isolated, for which the structures were assigned on the basis of ^1^H NMR spectra including COSY and NOESY measure-ments. These types of products are attractive starting materials for synthesis of potential anticancer PDT agents with unique structures, being practically unavailable by any other alternative method.

## 1. Introduction

A number of porphyrin derivatives are of significant importance due to their potential use in many fields of chemistry, medicine, pharmacology, new materials, etc. [1]. The desired precursors for the synthesis of these compounds can be isolated from naturally occurring substances (e.g., chlorophyll, heme) or prepared via transformations of simple synthetic moieties (e.g., *meso*-tetraphenylporphyrin, TPP). Thus, nowadays the selective functionalization of porphyrins is being studied intensively.

**Scheme 1:**
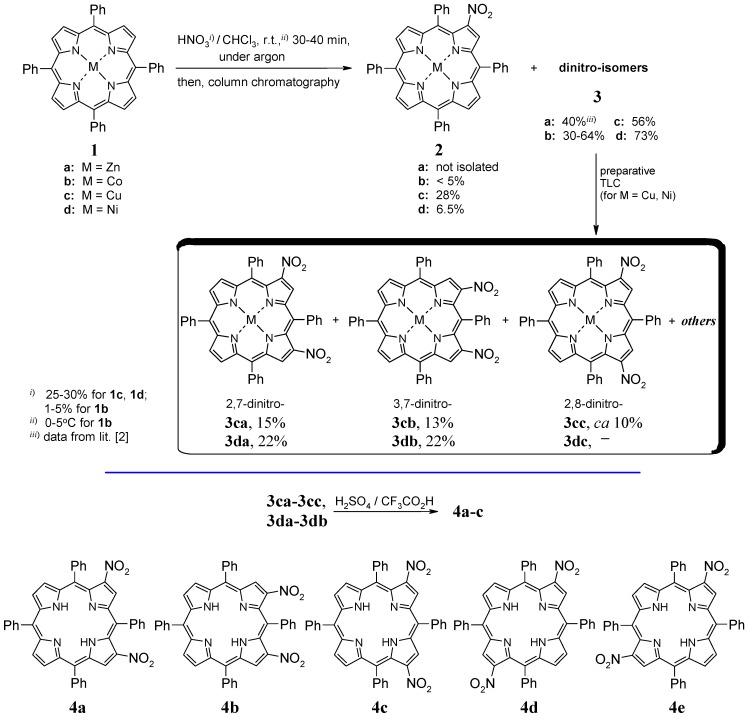
Preparation of dinitro-isomers and all the possible structures of the products.

Recently, successful nitration of TPP zinc complex **1a** (Scheme 1), leading mainly to [2,7-dinitro-5,10,15,20-tetra-phenylpor-phyrinato]zinc(II) as the major product with a satisfactory yield *ca* 40%, has been demonstrated [2]. The respective dinitroporphyrins of this type are valuable starting materials for our ongoing projects; e.g., (a) an exhaustive substitution at all *β*-positions in two neighbouring pyrrole rings (which allows the synthesis of highly substituted porphyrins) [3] or (b) controlled cycloaddition reactions leading to isobacteriochlorins [4], etc. They could also serve as interesting models for electrochemical studies [5].

Thus, we have attempted to elaborate the dinitration reaction for some additional TPP chelates (Cu^II^, Ni^II^, Co^II^), in search for other *β*,*β*-dinitro-isomers. Such a nitration of a copper complex has been already reported. Dahal et al. observed mixtures of two or three dinitro-compounds in various reactions [6]. However, the total yields were relatively low (*ca* 20%) and no information was provided about the yields of individual isolated isomers. The resulting NO_2_-disubstituted porphyrins could be very attractive intermediates for synthesis of the target moieties of higher complexity, e.g., potential anticancer agents [7,8,9,10,11].

## 2. Results and Discussion

Three TPP complexes (Cu^II^, Ni^II^, Co^II^) were used for our investigations. It is worth mentioning that mononitration (with good yields) of all these systems under electrophilic conditions (HNO_3_ in CHCl_3_) was described earlier [12,13]. Additionally zinc complex gave satisfactory results [2]. Unexpectedly, we discovered lately that some free base porphyrins can react according to this scheme, as well [14]. Finally, the *β*-monosubstituted products, contrary to polysubstituted ones, are readily available by various other methods [15,16,17,18,19,20].

### 2.1. Nitration of Cobalt(II) and Copper(II) Complexes

Initially, in the reactions of cobalt complex **1b** (under various conditions: 1–5% HNO_3_, 5 min, 2 h, 0–20 °C, in CHCl_3_, under argon) the formation of dinitro-compounds with yields in the 30–64% range was observed. However, the products formed included partial decomplexation derivatives, yielding practically inseparable mixtures. Thus, these experiments were temporarily suspended.

We found in our laboratory that copper complexes were the most convenient substrates for these transformations (the total nitration yield is high and the chromatographic isolation of the products is relatively satisfactory). Thus, the selected TPP copper(II) porphyrinate **1c** was subjected to the reaction with nitric acid in CHCl_3_, applying the method based on our previously reported procedures, elaborated for mononitration [12,13]. Initially, this electrophilic reaction with the use HNO_3_ of higher concentration (35–55%, freshly prepared from fuming yellow nitric acid) usually gave a mixture of dinitro-compounds, in 30–43% yield. These mixtures contained three (or more) *β*,*β*-dinitro-isomers: 2,7- (one spot on TLC) and 2,8-/3,7- (another spot on TLC) in a *ca* 1:1 ratio. We found that from these mixtures the two major dinitro-isomers—2,7-dinitro- (**3ca**) and 3,7-dinitro-5,10,15,20-tetraphenylporphyrin-copper(II) (**3cb**)—could be isolated in considerable amounts by precise column chromatography followed by preparative TLC (see Scheme 1 and Experimental). In the remaining chromatographic fractions some amounts of these isomers were still present. It should be also mentioned that in these reactions formation of the mononitro-product **2c** was observed, with variable yields (*ca* 10%).

In the case of this complex (**1c**), partial optimization of the reaction conditions allowed us to obtain the best yield of *β*,*β*-dinitro-isomers when using 25% HNO_3_. Thus, treatment of **1c** with the above nitric acid in CHCl_3_ at room temperature (reaction time—30–40 min) gave moderate amounts of monosubstituted product (**2c**, 28%) and a mixture of the desired dinitrated derivatives (56% combined, confirmed by MS measurements) (Scheme 1). The above fractions were isolated by column chromatography, however separation of the major dinitro-isomers required precise TLC conditions (CHCl_3_/*n*-hexane—1:1). In this reaction five different dinitro-isomers may form (Scheme 1). The investigations reported herein allowed us to identify the three main products.

Product **3ca** (15%) and its isomer **3cb** (13%) were obtained as analytically pure compounds. Additionally, a fraction containing (2,8-dinitro-5,10,15,20-tetra-phenylpor-phyrinato)copper(II) (**3cc**; contaminated with **3cb**) was isolated (*ca* 10%). It could be further purified by preparative TLC.

The corresponding structures of the products **3ca**–**3cc** couldn’t be assigned directly on the basis of NMR due to the paramagnetic properties of the copper cation, thus some of them were identified by comparison with authentic compounds obtained earlier in our laboratory as by-products of other reactions, or selected compounds that were demetallated. Removal of the paramagnetic copper cation allowed us to record ^1^H NMR spectra (see Supplementary Materials) and fully characterize the products as the free base porphyrins **4a**–**c**. This problem is discussed in details in Section 2.4. The demetallation reactions were carried out in a mixture of acids (CF_3_CO_2_H/H_2_SO_4_) at room temperature (15–30 min), leading to the metal-free derivatives in high yields (*ca* 90%, see Experimental).

### 2.2. Nitration of Nickel(II) Complex

In the case of nitration of the nickel complex **1d** (20–30% HNO_3_, 10–40 min) we also observed the formation of considerable amounts of a mixture of dinitro-derivatives (yield 18–64%), always accompanied by mono-substituted product. Partial optimization of the reaction conditions (a large excess of 30% HNO_3_/CHCl_3_, r.t., 30 min, under argon) allowed us to increase the dinitration product yield of up to 73% (mononitro-derivative: 6.5%, known compound **2d** [12]). It is worth mentioning that the yield was calculated for two steps (complexation of TPP and nitration) as the nickel complex **1d** is only moderately soluble; its purification by column chromatography is also somewhat troublesome, thus decreasing the isolated yield. The crude (*meso*-tetra-phenylpor-phyrinato)nickel(II) (**1d**) when directly subjected to the nitration reaction gave a mixture of several products from which only the 2-nitro-porphyrin **2d** was isolated easily by column chromatography, while a mixture of dinitro-compounds had to be separated additionally by preparative TLC (CHCl_3_/*n*-hexane—2:1, developed four times). From this mixture of dinitro-moieties two major isomers were isolated: (2,7-dinitro-*meso*-tetra-phenylpor-phyrinato)nickel(II) (**3da**, 22%) and (3,7-dinitro-*meso*-tetra-phenylpor-phyrinato)-nickel(II) (**3db**, 22%). ^1^H NMR, MS, HR-MS and UV-vis analyses confirmed their structures. In the ^1^H NMR of the 2,7-dinitro-compound **3da** there are two diagnostic downfield-shifted singlets of H^β^-protons neighbouring NO_2_ groups (8.89 ppm and 8.81 ppm) and two AB systems (in the region 8.56–8.69 ppm; from the remaining four *β*-protons). This is the only unsymmetrical *β*,*β*-dinitro–substituted product. Thus, its identification was very simple, contrary to assignment of the spectrum of compound **3db**. The latter case was not a trivial problem, and is discussed below (Section 2.4).

### 2.3. Nitration of 5,10,15,20-tetrakis(3-Methylphenyl)porphyrin–Copper(II) Complex (**5**)

**Figure 1:**
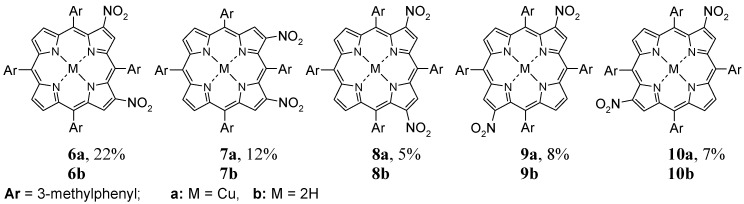
Structures of dinitro-isomers **6a**–**10a** and **6b–10b**.

The above substitution orientation and the yields concerning *β*,*β*-dinitration of TPP complexes should also be observed for other porphyrin derivatives. Indeed, this was the case. We selected [5,10,15,20-tetrakis(3-methylphenyl)porphyrinato]copper(II) (**5**) for this experiment and its treatment with the above nitric acid in CHCl_3_ gave similar results. Interestingly, all the possible dinitro-isomers **6a**–**10a** were successfully isolated (see Figure 1).

### 2.4. ^1^H NMR Spectra and Structure Elucidation of the Isomers

The correct assignment of the corresponding structures to the obtained products was not trivial. It was mainly accomplished on the basis of ^1^H NMR, however as the copper complexes used herein are paramagnetic their spectra are not recordable. On the other hand, Cu-chelates were the most convenient models for the studies (separation of the products was relatively satisfactory, yields were good, and three isomers were isolated). Thus, we solved this problem indirectly. From the mixture of dinitrated porphyrins three of them were isolated as pure individual compounds by preparative TLC. Their demetallation using H_2_SO_4_/CF_3_CO_2_H gave copper-free isomers, which were examined spectroscopically in details.

**Figure 2:**
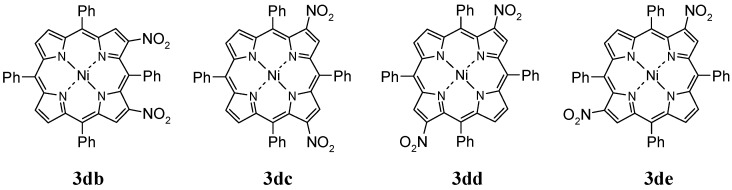
Structures of 3db, 3dc, 3dd, and 3de.

**Figure 3:**
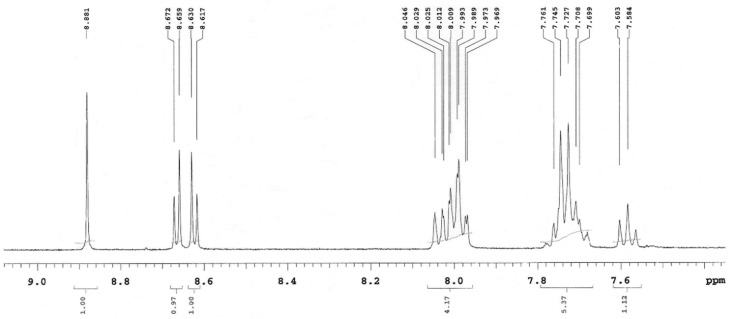
^1^H NMR spectrum of product **3db** (diagnostic region: 7.50–9.00 ppm).

**Figure 4:**
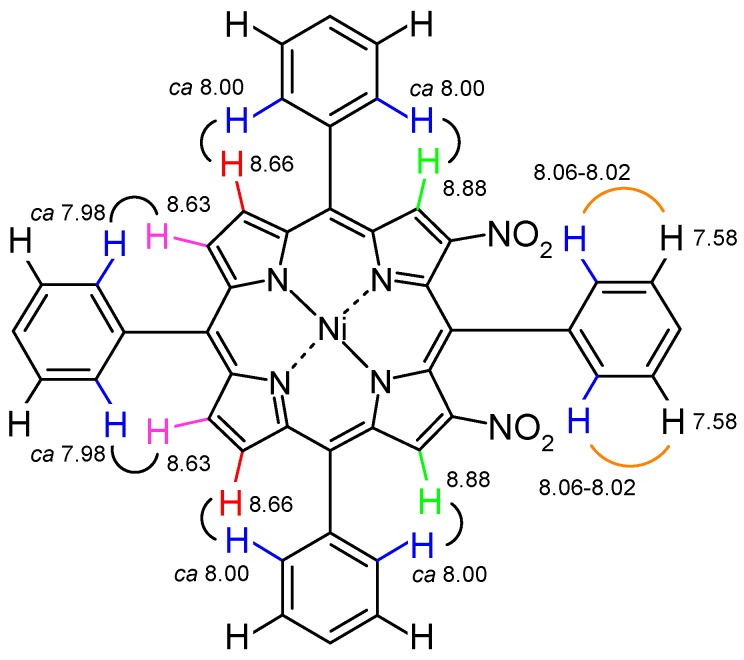
The diagnostic 2D correlations observed for product **3db**: COSY correlations—orange arches; NOESY correlations—black arches.

In the case of nickel products the isomers were analyzed as chelates. The identification of **3da** (2,7-dinitro-) was simple (see above) contrary to the assignment of the structure of the next isomer. In the spectrum of the latter we found a singlet δ = 8.88 ppm originating from two *β*-protons, and an AB-like system (4H) at 8.63 ppm and 8.66 ppm (with *J* = 5.2 Hz). Unfortunately, these data are in agreement with the three structures (**3db**, **3dc**, **3dd**; Figure 2). Structure **3de** was not taken under consideration because as a highly symmetrical one it should give in this region only three singlets (3 × 2H). On the basis of two-dimensional COSY and NOESY measurements structure of **3db** was assigned. There is a diagnostic correlation between the signals at 8.06–8.02 ppm (2H) and the triplet at 7.58 ppm (*J* = 7.6 Hz, 2H) (see Figure 3 and Figure 4). These signals are outside the two multi-H multiplets; thus, the corresponding protons must be under the strong influence of NO_2_ groups. We therefore ascribed them to the *ortho*- and *meta*-protons of one phenyl ring situated between two nitro groups. This is in agreement with the proposed 3,7-dinitro-structure **3db**. Moreover, in this analysis step the isomer **3dd** can be definitively excluded from the list of potential products because the integration ratio of all the *ortho*-protons of the *meso*-phenyl rings should be equal to 4:4 in this case.

Theoretically, the 2,8-dinitro-isomer **3dc** could also give such a correlation pattern. Thus, the structure **3db** was definitively confirmed by the NOESY technique. In the spectrum several correlations between protons due to their spatial proximity (Figure 4; black arches) were observed. The most diagnostic one is the correlation of the *ortho*-phenyl protons, δ = *ca* 8.00 ppm, with the two protons of the AB-like system (’the red protons’, at 8.66 ppm) from one side, and the two *β*-protons neighbouring the NO_2_ groups (’the green protons’, singlet at δ = 8.88 ppm), from the other side. Additionally, the *β*-protons of the remaining part of the AB-like system (2H, 8.63 ppm, ’the violet protons’) correlate with the *ortho*-protons of the last phenyl ring (δ = *ca* 7.98 ppm; in the 8.06–7.96 ppm multiplet). Finally, no correlation between the *ortho*-protons at 8.06–8.02 ppm with any *β*-protons was observed. This is an additional unambiguous evidence for the structure **3db**. Moreover, demetallation of **3db** and comparison of the ^1^H NMR spectrum of the free base porphyrin obtained with the data observed for **4b** (in the light of the information given below), allowed us to find the same 3,7-disubstitution pattern (the products were the same). By this way, we proved that the analyzed product, obtained in the nitration of TPP(Ni) (**1d**), has structure **3db**.

A similar analysis using 2D NMR techniques for a copper-free porphyrin **4b**, obtained from **3cb**, was performed earlier by Wyrębek [21]. After two-step transformation he isolated this product in 6% yield and its spectroscopic data were in good agreement with that described herein (see Experimental). In the case of the isomer **3ca** (obtained in our experiments), the identification based on the ^1^H NMR analysis of the demetallated porphyrin **4a** was similar to **3da**. In this series of compounds, again it was the only unsymmetrical *β*,*β*-dinitro–substituted product.

We also isolated the partially contaminated 2,8-dinitro-5,10,15,20-tetraphenylporphyrin-copper(II) (**3cc**). In ^1^H NMR spectrum of its free base form **4c** a very similar signal pattern was found. The key difference is the appearance of a signal for the four *β*-protons (H-12, H-13, H-17, H-18) as a singlet. Thus, also the structure of **3cc** was indirectly proved (via demetallation).

## 3. Experimental 

### 3.1. Materials and Methods—General.

^1^H NMR spectra were recorded with a GEMINI-200 or MR-400 spectrometers (both by Varian, Palo Alto, CA, USA) operating at 200 MHz and 400 MHz, respectively. Coupling constants *J* are expressed in Hertz [Hz]. Mass spectra were measured with a GCT Premier FD-TOF instrument (Waters, Milford, MA, USA) (FD method), a MARINER ESI-TOF spectrometer (ESI method, PerSeptive Biosystems, Framingham, MA, USA), and Synapt G2-S HDMS ESI-TOF spectrometer (ESI method) (Waters); *m/z* intensity values for peaks are given as % of relative intensity. UV-vis spectra were measured with a DU-68 (Beckman, Brea, CA, USA), SP-8001 (Metertech, Nangang, Taipei, Taiwan), UV-3600 (Shimadzu, Chiyoda-ku, Tokyo, Japan), and V-730 (Jasco, Hachioji, Tokyo, Japan) spectrophotometers. TLC analysis was performed on aluminium foil plates pre-coated with silica gel (60 F-254, Merck AG, Darmstadt, Germany). All the products were isolated by column chromatography (silica gel, 230–400 mesh; Merck AG). Some dinitro-isomers were additionally rechromatographed using preparative TLC plates (silica gel, 60 F-254, 2 mm and 0.5 mm; Merck AG). Molecular formulas of new compounds were confirmed by HR-MS (ESI, EI, and FD). Starting porphyrinates were obtained according to known procedures described in the previous literature [12,13]. Some dinitro-derivatives were also reported. Their ^1^H NMR, UV-vis, and MS spectra are in agreement with the spectra described herein. These spectroscopic data are given below for more detailed and accurate characterization of the products.

### 3.2. Procedures and Data of New Compounds

#### 3.2.1. Nitration of Copper(II) Complex of 5,10,15,20-Tetraphenylporphyrin (**1c**)

To a stirred solution of (*meso*-tetra-phenylpor-phyrinato)copper(II) (**1c**; 237 mg, 0.35 mmol) in CHCl_3_ (530 mL) at room temperature, a solution of 25% aqueous nitric acid (freshly prepared from fuming yellow HNO_3_, *d* = 1.52; large excess, 140 mL, 637 mmol) was added dropwise during *ca* 5 min. The reaction mixture was intensively stirred under argon in a round-bottomed flask, protected against light, for 30–40 min with TLC monitoring (CHCl_3_/*n*-hexane—1:1). Then, the mixture was poured into aqueous solution of 5% NaHCO_3_ (200 mL), and shaken carefully in a separatory funnel. The separated organic layer was washed with water (4 × 200 mL), and dried with anhydrous MgSO_4_/Na_2_CO_3_. After evaporating the solvent, the residue was subjected to column chromatography (eluent: CHCl_3_/*n*-hexane—1:1) to give (2-nitro-5,10,15,20-tetra-phenylpor-phyrinato)copper(II) (**2c**; 71 mg, 28%) and a mixture of dinitro-substituted isomers (150 mg, 56%). The dinitro-isomers were separated on preparative TLC (CHCl_3_/*n*-hexane—1:1, four times developed), allowing isolation of: (a) (2,7-dinitro-5,10,15,20-tetra-phenylpor-phyrinato)copper(II) (**3ca**; 40 mg, 15%); (b) (3,7-dinitro-5,10,15,20-tetra-phenylpor-phyrinato)copper(II) (**3cb**; 35.5 mg, 13%); (c) (2,8-dinitro-5,10,15,20-tetra-phenylpor-phyrinato)copper(II) (**3cc**) contaminated with small amounts of (3,7-dinitro-5,10,15,20-tetra-phenylpor-phyrinato)copper(II) (**3cb**) (30 mg, yield—*ca* 10%). **3cc** can be further purified by preparative TLC.

It was reported earlier that when 50% HNO_3_ was used (temp. 5–10 °C, reaction time—6 min, the post-reaction mixture was poured into water with ice, followed by column chromatography: CHCl_3_/*n*-hexane—from 1:1 to 2:1), two major isomers were isolated: 2,7-dinitro- (**3ca**) and 3,7-dinitro-5,10,15,20-tetraphenylporphyrin-copper(II) (**3cb**) [21]. However, the combined yield was consider-ably lower (20%). Additionally, a mixture of the remaining dinitro-isomers was isolated (23%).

##### Products Data 

*(2-Nitro-5,10,15,20-tetra-phenylpor-phyrinato)copper(II)* (**2c**): known compound [12,15,16,17].

*(2,7-Dinitro-5,10,15,20-tetra-phenylpor-phyrinato)copper(II)* (**3ca**): m.p. > 300 °C. UV-vis (CHCl_3_); λ_max_ [nm] (log ε): 559 (3.58), 438 (4.55, Soret band). MS (ESI); *m/z* (% rel. int.): 769 (14), 768 (42), 767 (78), 766 (81), 765 (100) [isotope M**^+•^** and (M + H)^+^]. HR-MS (ESI): *m/z* calcd for C_44_H_26_N_6_O_4_Cu [M**^+^**]: 765.1312; found: 765.1327. MS (FD), *m/z* (% rel. int.): 769 (8), 768 (24), 767 (58), 766 (49), 765 (100) [isotope M**^+•^**]. HR-MS (FD): *m/z* calcd for C_44_H_26_N_6_O_4_Cu [M**^+^**]: 765.1312; found: 765.1344.

*(3,7-Dinitro-5,10,15,20-tetra-phenylpor-phyrinato)copper(II)* (**3cb**): m.p. > 300 °C. UV-vis (CHCl_3_); λ_max_ [nm] (log ε): 607 (4.02), 560.5 (4.19), 437.5 (5.25, Soret band), 388 (4.52), 326 (4.44). MS (FD), *m/z* (% rel. int.): 769 (7), 768 (24), 767 (58), 766 (49), 765 (100) [isotope M**^+•^**]. HR-MS (FD): *m/z* calcd for C_44_H_26_N_6_O_4_Cu [M**^+^**]: 765.1312; found: 765.1327.

*(2,8-Dinitro-5,10,15,20-tetra-phenylpor-phyrinato)copper(II)* (**3cc**): m.p. > 300 °C. UV-vis (CHCl_3_); λ_max_ [nm] (log ε): 601.5 (4.21), 561 (4.43), 436.5 (5.45, Soret band). MS (ESI); *m/z* (% rel. int.): 769 (17), 768 (38), 767 (61), 766 (79), 765 (100) [isotope M**^+•^** and (M + H)**^+^**]. HR-MS (ESI): *m/z* calcd for C_44_H_26_N_6_O_4_Cu [M**^+^**]: 765.1312; found: 765.1260.

The above products were also fully characterized as their corresponding decomplexed forms **4a**–**c** (see below).

#### 3.2.2. Nitration of Nickel(II) Complex of 5,10,15,20-Tetraphenylporphyrin (**1d**)

The substrate, (*meso*-tetra-phenylpor-phyrinato)nickel(II) (**1d**), was prepared from 0.11 mmol of TPP according to procedure described earlier for a similar compound [13], and it was directly subjected to nitration reaction. This crude complex was dissolved in CHCl_3_ (200 mL) and to a stirred solution obtained at room temperature, a solution of 30% aqueous nitric acid (freshly prepared from yellow HNO_3_, *d* = 1.52; 13.8 mL, 77.5 mmol) was added dropwise via syringe (using septum inlet) during *ca* 5 min. The reaction was carried out with intensive stirring in a round-bottomed flask, protected against light, under argon, for 30 min. Then, the mixture was poured into 5% aqueous solution of NaHCO_3_ (150 mL), and shaken carefully in a separatory funnel. The separated organic layer was washed with water (4 × 150 mL) and dried with anhydrous MgSO_4_/Na_2_CO_3_. After evaporating the solvent, the residue was subjected to column chromatography (eluent: CHCl_3_/*n*-hexane—2:1) to give (2-nitro-5,10,15,20-tetra-phenylpor-phyrinato)nickel(II) (**2d**; 5.0 mg, 6.5%) and a mixture of dinitro–substituted compounds (61 mg, 73%). The dinitro-isomers were separated by preparative TLC (CHCl_3_/*n*-hexane—2:1, developed four times), thus allowing isolation of: (a) (3,7-dinitro-5,10,15,20-tetra-phenylpor-phyrinato)nickel(II) (**3db**; 18.4 mg, 22%); (b) (2,7-dinitro-5,10,15,20-tetra-phenylpor-phyrinato)nickel(II) (**3da**; 18.4 mg, 22%); (c) an inseparable mixture of the remaining dinitro-isomers (22 mg, 26%). All the above yields are calculated for two steps.

##### Products Data 

*(2-Nitro-5,10,15,20-tetra-phenylpor-phyrinato)nickel(II)* (**2d**): known compound; it has been already described in our previous paper [12].

*(2,7-Dinitro-5,10,15,20-tetra-phenylpor-phyrinato)nickel(II)* (**3da**): m.p. > 300 °C. ^1^H NMR (CDCl_3_, 400 MHz); δ [ppm]: 8.89 (s, 1H, H^β^-pyrrole), 8.81 (s, 1H, H^β^-pyrrole), 8.69 and 8.58 (AB system, *J* = 5.1 Hz, 2H, H^β^-pyrrole), 8.64 and 8.56 (AB system, *J* = 5.1 Hz, 2H, H^β^-pyrrole), 8.02–7.95 (m, 8H, H-Ph), 7.76–7.62 (m, 12H, H-Ph). UV-vis (CHCl_3_); λ_max_ [nm]: 556.5, 449 (Soret band), 389.5, 329. MS (FD), *m/z* (% rel. int.): 765 (4), 764 (12), 763 (23), 762 (50), 761 (51), 760 (100) [isotope M**^+•^**]. The above data were in agreement with those described earlier [12].

*(3,7-Dinitro-5,10,15,20-tetra-phenylpor-phyrinato)nickel(II)* (**3db**): m.p. > 300 °C. ^1^H NMR (CDCl_3_, 400 MHz); δ [ppm]: 8.88 (s, 2H, H^β^-pyrrole), 8.66 and 8.63 (AB-like system, *J* = 5.2 Hz, 4H, H^β^-pyrrole), 8.06–7.96 (m, 8H, H-Ph), 7.78–7.68 (m, 10H, H-Ph), 7.58 (t, *J* = 7.6 Hz, 2H, H-Ph). UV-vis (CHCl_3_); λ_max_ [nm]: 596, 553, 446 (Soret band), 382, 319. MS (FD), *m/z* (% rel. int.): 765 (4), 764 (12), 763 (22), 762 (50), 761 (53), 760 (100) [isotope M**^+•^**]. HR-MS (ESI): *m/z* calcd for C_44_H_26_N_6_O_4_Ni [M**^+^**]: 760.1369; found: 760.1403.

#### 3.2.3. Demetallation

Demetallation reactions to give free base porphyrins were carried out according to modified known procedures [12,13,22]: a) Cu-complexes (CF_3_CO_2_H/H_2_SO_4_, 1:1; r.t.; 15–30 min); yields: **4a** from **3ca**, 83%; **4b** from **3cb**, 93%; **4c** from **3cc**, 61%; b) Ni-complex (under conditions as above); yield of **4b** from **3db**, 80%. The analytically pure products were fully characterized and the spectra confirmed the structures. The compounds obtained from **3cb** and from **3db** exhibited identical spectral properties as assigned to compound **4b**.

##### Products Data 

*2,7-Dinitro-5,10,15,20-tetraphenylporphyrin* (**4a**): m.p. > 300 °C. ^1^H NMR (CDCl_3_, 200 MHz); δ [ppm]: 9.04 (s, 1H, H^β^-pyrrole), 9.03 (s, 1H, H^β^-pyrrole), 8.84 and 8.75 (AB system, *J* = 5.0 Hz, 2H, H^β^-pyrrole), 8.79 and 8.75 (AB system, *J* = 5.0 Hz, 2H, H^β^-pyrrole), 8.28–8.15 (m, 8H, H-Ph), 7.84–7.68 (m, 12H, H-Ph), −2.17 (s, 2H, 2 × NH). UV-vis (CHCl_3_); λ_max_ [nm] (log ε): 685 (3.42), 538.5 (3.55), 438 (4.68, Soret band). MS (FD), *m/z* (% rel. int.): 707 (3), 706 (11), 705 (49), 704 (100) [isotope M**^+•^**]. HR-MS (EI): *m/z* calcd for C_44_H_28_N_6_O_4_ [M**^+^**]: 704.2172; found: 704.2182.

*3,7-Dinitro-5,10,15,20-tetraphenylporphyrin* (**4b**): m.p. > 300 °C. ^1^H NMR (CDCl_3_, 400 MHz); δ [ppm]: 8.97 (s, 2H, H^β^-pyrrole), 8.79 and 8.76 (AB-like system, *J* = 5.0 Hz, 4H, H^β^-pyrrole), 8.29–8.26 (m, 2H, H-Ph), 8.22–8.15 (m, 6H, H-Ph), 7.86–7.75 (m, 10H, H-Ph), 7.67 (t, *J* = 7.6 Hz, 2H, H-Ph), −2.25 (s, 2H, 2 × NH). UV-vis (CHCl_3_); λ_max_ [nm] (log ε): 685 (3.76), 584 (3.92), 540 (3.90), 444.5 (5.09, Soret band). MS (FD), *m/z* (% rel. int.): 707 (4), 706 (14), 705 (52), 704 (100) [isotope M**^+•^**]. HR-MS (EI): *m/z* calcd for C_44_H_28_N_6_O_4_ [M**^+^**]: 704.2172; found: 704.2149.

*2,8-Dinitro-5,10,15,20-tetraphenylporphyrin* (**4c**): m.p. > 300 °C. ^1^H NMR (CDCl_3_, 200 MHz); δ [ppm]: 9.01 (s, 2H, H^β^-pyrrole), 8.93 (s, 4H, H^β^-pyrrole), 8.32–8.15 (m, 8H, H-Ph), 7.87–7.69 (m, 12H, H-Ph), −2.49 and −2.58 (2 × broad s, 2H, 2 × NH). UV-vis (CHCl_3_); λ_max_ [nm]: 682, 537.5, 434 (Soret band). MS (ESI); *m/z* (% rel. int.): 707 (13), 706 (52), 705 (100), 704 (7) [isotope M**^+•^** and (M + H)^+^]. HR-MS (ESI): *m/z* calcd for C_44_H_29_N_6_O_4_ [(M + H)**^+^**]: 705.2250; found: 705.2276.

In the previous paper [12] the structures for the 3,7- and 2,8-isomers **4b** and **4c**, obtained in small amounts as by-products, were inversely assigned.

#### 3.2.4. Nitration of Copper(II) Complex of 5,10,15,20-tetrakis(3-Methylphenyl)porphyrin (**5**)

In a round-bottomed flask, to a stirred solution of complex **5** (102.4 mg, 0.140 mmol) in CHCl_3_ (130 mL), a solution of 33% aqueous nitric acid (freshly prepared from yellow HNO_3_, *d* = 1.52; 37.8 mL, 222 mmol) was added dropwise via syringe (using septum inlet) during *ca* 15 min, under argon, at room temperature. The reaction was vigorously stirred for additional 10 min. Then, the mixture was washed with water (4 × 70 mL), and the combined water layers were extracted with CHCl_3_ (25 mL). The combined organic layers were dried over anhydrous MgSO_4_/Na_2_CO_3_. After evaporating the solvent, the residue was subjected to column chromatography (CHCl_3_/*n*-hexane—1:1) to give: (a) [2,7-dinitro-5,10,15,20-tetrakis(3-methylphenyl)porphyrinato]copper(II) (**6a**; 25.4 mg, 22%); (b) [3,7-dinitro-5,10,15,20-tetrakis(3-methylphenyl)porphyrinato]copper(II) (**7a**; 13.8 mg, 12%); (c) [2,8-dinitro-5,10,15,20-tetrakis(3-methylphenyl)porphyrinato]copper(II) (**8a**; 5.4 mg, 5%); (d) [2,12-dinitro-5,10,15,20-tetrakis(3-methylphenyl)porphyrinato]copper(II) (**9a**; 8.9 mg, 8%); (e) [2,13-dinitro-5,10,15,20-tetrakis(3-methylphenyl)porphyrinato]copper(II) (**10a**; 8.3 mg, 7%).

In some experiments traces of trinitro-isomers were detected (*ca* 1%, identified by MS). The structures of the above compounds could not be elucidated due to the paramagnetic copper cation inside the core ring, thus their ^1^H NMR spectra are not readable. Additionally, the compounds **8a**,**9a** were isolated as an inseparable mixture, so to determine the structures, all the compounds obtained and the mixture **8a**/**9a** were demetallated [12,13,22] to give free base porphyrins **6b**–**10b** via column chromatography (eluent: CHCl_3_/*n*-hexane). The spectral data of the products **6a**–**10a** are given below. Also the porphyrins **6b**–**10b** were fully characterized. The yield of **8a** and **9a** was calculated on the basis of the ratio of the separated **8b** and **9b**.

##### Products Data

*[2,7-Dinitro-5,10,15,20-tetrakis(3-methylphenyl)porphyrinato]copper(II)* (**6a**): m.p. > 300 °C. UV-vis (CHCl_3_); λ_max_ [nm]: 606, 561.5, 440.5 (Soret band), 313. MS (ESI); *m/z* (% rel. int.): 847 (3), 846 (7), 845 (8), 844 (14) [isotope (M + Na)**^+^**], 825 (8), 824 (26), 823 (55), 822 (58), 821 (100) [isotope M**^+^^•^**]. HR-MS (ESI): *m/z* calcd for C_48_H_34_N_6_O_4_Cu [M**^+^**]: 821.1938; found: 821.1925.

*[3,7-Dinitro-5,10,15,20-tetrakis(3-methylphenyl)porphyrinato]copper(II)* (**7a**): m.p. > 300 °C. UV-vis (CHCl_3_); λ_max_ [nm] (log ε): 608.5 (3.53), 561.5 (3.67), 441.5 (4.80, Soret band), 271.5 (4.30). MS (ESI); *m/z* (% rel. int.): 847 (7), 846 (14), 845 (14), 844 (25) [isotope (M + Na)**^+^**], 825 (9), 824 (29), 823 (61), 822 (63), 821 (100) [isotope M**^+^^•^**]. HR-MS (ESI): *m/z* calcd for C_48_H_34_N_6_O_4_Cu [M**^+^**]: 821.1938; found: 821.1924.

*[2,8-Dinitro-5,10,15,20-tetrakis(3-methylphenyl)porphyrinato]copper(II)* (**8a**) and [2,12-dinitro-5,10,15,20-tetrakis(3-methylphenyl)porphyrinato]copper(II) (**9a**); inseparable mixture: MS (ESI); m/z (% rel. int.): 847 (6), 846 (13), 845 (12), 844 (23) [isotope (M + Na)^+^], 825 (8), 824 (29), 823 (58), 822 (59), 821 (100) [isotope M^+^^•^].

*[2,13-Dinitro-5,10,15,20-tetrakis(3-methylphenyl)porphyrinato]copper(II)* (**10a**): m.p. > 300 °C. UV-vis (CHCl_3_); λ_max_ [nm]: 606, 562.5, 435.5 (Soret band). MS (ESI); *m/z* (% rel. int.): 847 (6), 846 (12), 845 (12), 844 (23) [isotope (M + Na)^+^], 825 (8), 824 (29), 823 (58), 822 (59), 821 (100) [isotope M**^+^^•^**]. HR-MS (ESI): *m/z* calcd for C_48_H_34_N_6_O_4_Cu [M**^+^**]: 821.1938; found: 821.1925.

*2,7-Dinitro-5,10,15,20-tetrakis(3-methylphenyl)porphyrin* (**6b**): m.p. > 300 °C. ^1^H NMR (CDCl_3_, 400 MHz); δ [ppm]: 9.05 (s, 1H, H^β^-pyrrole), 9.03 (s, 1H, H^β^-pyrrole), 8.86 (d, *J* = 5.1 Hz, 1H, H^β^-pyrrole), 8.78 and 8.75 (AB system, *J* = 4.7 Hz, 2H, H^β^-pyrrole), 8.74 (d, *J* = 5.1 Hz, 1H, H^β^-pyrrole), 8.08–7.95 (m, 8H, H-Ar), 7.69–7.56 (m, 8H, H-Ar), 2.66 (s, 6H, 2 × CH_3_), 2.64 and 2.63 (2 × s, 6H, 2 × CH_3_), −2.22 (s, 2H, 2 × NH). UV-vis (CHCl_3_); λ_max_ [nm]: 683, 582, 540, 444 (Soret band), 366. MS (ESI); *m/z* (% rel. int.): 764 (2), 763 (11), 762 (48), 761 (100) [isotope (M + H)^+^]. HR-MS (ESI): *m/z* calcd for C_48_H_37_N_6_O_4_ [(M + H)**^+^**]: 761.2876; found: 761.2855.

*3,7-Dinitro-5,10,15,20-tetrakis(3-methylphenyl)porphyrin* (**7b**): m.p. > 300 °C. ^1^H NMR (CDCl_3_, 400 MHz); δ [ppm]: 8.98 (s, 2H, H^β^-pyrrole), 8.79 and 8.76 (AB-like system, *J* = 4.9 Hz, 4H, H^β^-pyrrole), 8.11–7.95 (m, 8H, H-Ar), 7.69–7.52 (m, 8H, H-Ar), 2.67 (s, 6H, 2 × CH_3_), 2.65 (s, 3H, CH_3_), 2.61 (s, 3H, CH_3_), −2.28 (s, 2H, 2 × NH). UV-vis (CHCl_3_); λ_max_ [nm] (log ε): 684 (3.86), 587.5 (4.02), 539 (3.97), 446 (5.21, Soret band), 396.5 (4.49). MS (ESI); *m/z* (% rel. int.): 764 (2), 763 (11), 762 (45), 761 (100) [isotope (M + H)^+^]. HR-MS (ESI): *m/z* calcd for C_48_H_37_N_6_O_4_ [(M + H)**^+^**]: 761.2876; found: 761.2855.

*2,8-Dinitro-5,10,15,20-tetrakis(3-methylphenyl)porphyrin* (**8b**): m.p. > 300 °C. ^1^H NMR (CDCl_3_, 400 MHz); δ [ppm]: 9.06 (s, 2H, H^β^-pyrrole), 8.85 (d, *J* = 4.7 Hz, 2H, H^β^-pyrrole), 8.73 (d, *J* = 4.7 Hz, 2H, H^β^-pyrrole), 8.06–7.95 (m, 8H, H-Ar), 7.68–7.55 (m, 8H, H-Ar), 2.67 (s, 6H, 2 × CH_3_), 2.62 (s, 6H, 2 × CH_3_), NH—undetected. UV-vis (CHCl_3_); λ_max_ [nm] (log ε): 679 (3.83), 580.5 (4.00), 538 (4.08), 439.5 (5.26, Soret band), 363.5 (4.44), 328 (4.37). MS (ESI); *m/z* (% rel. int.): 764 (2), 763 (13), 762 (48), 761 (100) [isotope (M + H)^+^]. HR-MS (ESI): *m/z* calcd for C_48_H_37_N_6_O_4_ [(M + H)**^+^**]: 761.2876; found: 761.2860.

*2,12-Dinitro-5,10,15,20-tetrakis(3-methylphenyl)porphyrin* (**9b**): m.p. > 300 °C. ^1^H NMR (CDCl_3_, 400 MHz); δ [ppm]: 9.02 and 8.92 (AA’XX’, *J*_AX_ = 5.0 Hz, 4H, H^β^-pyrrole), 9.01 (s, 2H, H^β^-pyrrole), 8.11–7.98 (m, 8H, H-Ar), 7.70–7.57 (m, 8H, H-Ar), 2.68 (s, 6H, 2 × CH_3_), 2.63 (s, 6H, 2 × CH_3_), −2.61 (s, 2H, 2 × NH). UV-vis (CHCl_3_); λ_max_ [nm] (log ε): 685 (3.72), 538 (3.73), 436 (4.97, Soret band), 361.5 (4.01). MS (ESI); *m/z* (% rel. int.): 764 (1), 763 (8), 762 (38), 761 (100), 760 (16) [isotope M**^+•^** and (M + H)**^+^**]. HR-MS (ESI): *m/z* calcd for C_48_H_37_N_6_O_4_ [(M + H)**^+^**]: 761.2876; found: 761.2862.

*2,13-Dinitro-5,10,15,20-tetrakis(3-methylphenyl)porphyrin* (**10b**): m.p. > 300 °C. ^1^H NMR (CDCl_3_, 400 MHz); δ [ppm]: 9.02 (s, 2H, H^β^-pyrrole), 8.97 (s, 2H, H^β^-pyrrole), 8.92 (s, 2H, H^β^-pyrrole), 8.12–7.97 (m, 8H, H-Ar), 7.69–7.57 (m, 8H, H-Ar), 2.67 (s, 6H, 2 × CH_3_), 2.64 (s, 6H, 2 × CH_3_), −2.50 (s, 1H, NH), −2.59 (s, 1H, NH). UV-vis (CHCl_3_); λ_max_ [nm]: 688.5, 539.5, 441 (Soret band), 370, 276.5. MS (ESI); *m/z* (% rel. int.): 785 (1), 784 (4), 783 (8) [isotope (M + Na)**^+^**], 764 (2), 763 (12), 762 (46), 761 (100), 760 (9) [isotope M**^+•^** and (M + H)**^+^**]. HR-MS (ESI): *m/z* calcd for C_48_H_37_N_6_O_4_ [(M + H)**^+^**]: 761.2876; found: 761.2870.

## 4. Conclusions

We have described herein the preparation and isolation of dinitro-isomers of TPP complexes (and their free base derivatives), which were formed in electrophilic nitration reactions of the parent compounds. Some of these isomers were reported in previous papers, however they were formed in low yields [6] (or as by-products [12]), and were not characterized in details or sometimes were characterized erroneously (e.g., the ^1^H NMR spectra) [6,12].

In the above reactions the formation of five dinitro-isomers is possible. Usually, we could isolate three of them (except for [5,10,15,20-tetrakis(3-methylphenyl)porphyrinato]copper(II)). In the case of copper complexes, the products were treated with H_2_SO_4_/CF_3_CO_2_H mixture, thus giving metal-free porphyrins that were also fully characterized. This type of porphyrins are practically not available by alternative methods. The synthesis of potential anticancer PDT agents derived therefrom by our group will be reported soon. 

## Supplementary Materials

The following are available online. Figures S1–S56 (^1^H NMR, UV-vis, and MS spectra of all the products).
